# Tau Accumulation in the Spinal Cord Contributes to Chronic Inflammatory Pain by Upregulation of IL-1β and BDNF

**DOI:** 10.1007/s12264-023-01152-4

**Published:** 2023-12-26

**Authors:** Shuxia Zhang, Yeru Chen, Yongjie Wang, Hongwei Wang, Dandan Yao, Gang Chen

**Affiliations:** 1 Department of Anesthesiology, School of Medicine, Sir Run Run Shaw Hospital, Zhejiang University, Hangzhou, 310016 China; 2 Key Laboratory of Elemene Anti-Cancer Medicine of Zhejiang Province and Holistic Integrative Pharmacy Institutes, Hangzhou Normal University, Hangzhou, 311121 China; 3 Engineering Laboratory of Development and Application of Traditional Chinese Medicine from Zhejiang Province, Holistic Integrative Pharmacy Institutes, Hangzhou Normal University, Hangzhou, 311121 China

**Keywords:** Tau, Inflammatory pain, IL-1β, BDNF

## Abstract

Microtubule-associated protein Tau is responsible for the stabilization of neuronal microtubules under normal physiological conditions. Much attention has been focused on Tau’s contribution to cognition, but little research has explored its role in emotions such as pain, anxiety, and depression. In the current study, we found a significant increase in the levels of p-Tau (Thr231), total Tau, IL-1β, and brain-derived neurotrophic factor (BDNF) on day 7 after complete Freund's adjuvant (CFA) injection; they were present in the vast majority of neurons in the spinal dorsal horn. Microinjection of *Mapt*-shRNA recombinant adeno-associated virus into the spinal dorsal cord alleviated CFA-induced inflammatory pain and inhibited CFA-induced IL-1β and BDNF upregulation. Importantly, Tau overexpression was sufficient to induce hyperalgesia by increasing the expression of IL-1β and BDNF. Furthermore, the activation of glycogen synthase kinase 3 beta partly contributed to Tau accumulation. These findings suggest that Tau in the dorsal horn could be a promising target for chronic inflammatory pain therapy.

**Supplementary Information:**

The online version contains supplementary material available at 10.1007/s12264-023-01152-4.

## Introduction

Pain is the fifth vital sign following body temperature, pulse, respiration, and blood pressure [1]. Unlike acute pain, which serves as a “warning”, persistent pain such as chronic inflammatory pain degrades physical and mental health and quality of life. Depression is diagnosed in up to 66% and anxiety in up to 70% of rheumatoid arthritis patients with chronic inflammatory pain [2]. Inflammatory pain is usually triggered by peripheral tissue injury and inflammation and is commonly treated in the clinic with anti-inflammatory and analgesic drugs. However, drug treatment is often accompanied by multiple adverse effects, including gastrointestinal discomfort and cardiovascular events [3, 4]. Thus, further research into the underlying mechanism of inflammatory pain is essential for the development of potential new drug treatments.

Tau is responsible for stabilizing neuronal microtubules under normal physiological conditions. In pathological situations, the accumulation of hyperphosphorylated Tau is a crucial cause of Alzheimer's disease (AD), and hyperphosphorylated Tau also accumulates in the spinal cord of AD patients [5]. In addition, some AD patients have hyperalgesia, and chronic pain can also increase the incidence of AD [6–8]. A study reported that, compared with wild-type (*Tau*^+/+^) mice, the response of *Tau* knockout (KO) mice to acute noxious stimuli decreased, whereas their pain-related behaviors were augmented under tonic inflammatory pain stimulation [9, 10]. Another study found that an excessive association of Tau protein with microtubules is capable of affecting neurogenesis, thereby impairing pain sensitivity [11]. All of these findings suggest that there might be a certain relationship between Tau and pain. However, it is unclear whether and how abnormal Tau expression mediates pain in a specific region.

Neuroinflammation is involved in the development of pain in the spinal dorsal horn by modulating neuronal excitability and affecting synaptic plasticity [12, 13]. Inflammatory stimuli in rodent hind paws are sufficient to activate peripheral Aδ and C nociceptors and cause primary neurons to release various molecular mediators into the spinal dorsal cord, such as glutamate, substance P, adenosine triphosphate (ATP), chemokine ligand 2 (CCL2), tumor necrosis factor-alpha (TNFα), colony-stimulating factor-1 (CSF-1), and brain-derived neurotrophic factor (BDNF) [14]. These mediators directly activate second-order neurons, microglia, and astrocytes, triggering neuroinflammation and second-order neuron hyperexcitability. Studies have demonstrated that hyperphosphorylated pathological Tau induces microglial/astrocytic activation and neuroinflammation [15–17]. Therefore, we hypothesized that excessive accumulation of Tau could promote chronic inflammatory pain through neuroinflammation.

In the present study, mouse paws were injected with Complete Freund's Adjuvant (CFA) to induce chronic inflammatory pain. The phosphorylated proteomics technique revealed that the threonine site 231 of Tau was hyperphosphorylated in spinal L4–6 after CFA injection. In CFA-induced inflammatory pain, the accumulation of Tau was found to increase the inflammatory factors IL-1β and BDNF in the spinal cord, leading to pain hypersensitization. Furthermore, activation of glycogen synthase kinase 3 beta (GSK3B) was found to result in Tau upregulation and to mediate pain behaviors.

## Materials and Methods

### Animals

We used 6- to 8-week-old adult male C57BL/6 mice. All mice were housed at a comfortable temperature (22 °C–25 °C) with *ad libitum* access to water and food under a 12-h light/dark cycle (lights on from 07:00 to 19:00). All animal protocols were approved by the Animal Care and Use Committee of Zhejiang University of China (No. ZJU20220441). Efforts were made to minimize the number of animals used.

### Inflammatory Pain Model

To induce inflammation pain, 10 μL CFA (1:1 dilution with sterile saline, Sigma, F5881, Billerica, MA, USA) was injected into the left hind paw of mice using a 10-μL syringe under 3% isoflurane anesthesia [18]. The needle was held for 5 s to avoid leakage. 10 μL sterile saline (0.9% NaCl) was injected as a control.

### Tandem Mass Tag (*TMT*)-Based Phosphoproteomics Sequencing

To obtain sufficient tissue samples, we used Sprague−Dawley rats at 7 weeks old and injected 100 μL of CFA (1:1 dilution with sterile saline) or saline into their footpads. The L4–6 segment on day 7 following CFA injection was harvested after anesthesia. The extracted tissue was homogenized with sonication in 200 μL of ice-cold RIPA lysis buffer containing protease and phosphoprotease inhibitors and then centrifuged at 10,000 r/min for 10 min at 4 °C. The supernatant was collected as a protein sample. Protein concentrations were determined with a BCA Protein Assay Kit (Thermo Fisher Scientific). 100 mmol/L triethylammonium bicarbonate (TEAB; Thermo Fisher Scientific) was added to 100 μg of protein sample to a final volume of 100 μL. Approximately 5 μL of 200 mmol/L tris-(2-carboxyethyl) phosphine (Thermo Fisher Scientific) was added to the sample and incubated at 55 °C for 1 h. About 9 mg of iodoacetamide (IAA; Thermo Fisher Scientific) was dissolved with 132 μL of 100 mmol/L TEAB to obtain 375 mmol/L IAA. Approximately 5 μL of 375 mM IAA was then added to the sample and incubated in the dark for 30 min at room temperature. The proteins were precipitated by adding 6 volumes (~600 μL) of precooled (− 20 °C) acetone (Sigma-Aldrich) and stored at − 20 °C for at least 4 h. The sample was then centrifuged at 8,000 r/min for 10 min at 4 °C. The acetone was discarded, while the white precipitate was retained. The precipitate was allowed to dry for 2–3 min. The protein precipitate was resuspended in 100 μL of 50 mmol/L TEAB. 2.5 μg trypsin (Promega) was added, and the sample was then digested at 37 °C overnight. The TMT reagent (iTRAQ Reagent-8Plex Multiplex Kit, AB Sciex) was dissolved in 41 μL of anhydrous acetonitrile (Fisher Chemical). TMT labeling was performed by transferring the digestion solution to the TMT reagent and incubating it at room temperature for 1 h. About 8 μL of 5% hydroxylamine (Thermo Fisher Scientific) was added to the sample and incubated for 15 min to quench the reaction. Each iTRAQ-labeled digestion solution was subjected to high pH fractionation with the Pierce™ High pH Reversed-Phase kit (Thermo Fisher Scientific). The phosphorylated peptides were enriched with High-Select™ Fe-NTA Phosphopeptide Enrichment Kit (Thermo Fisher Scientific). The enriched phosphorylated peptide fragments were dissolved in the sample solution (0.1% formic acid and 2% acetonitrile), thoroughly shaken, and centrifuged (13,200 r/min, 10 min, and 4 °C). The supernatant was subjected to liquid chromatography-tandem mass spectrometry (LC-MS) procedures. The mobile phase A comprised 0.1% formic acid (FA; Sigma-Aldrich) and 2% acetonitrile (ACN; Fisher Chemical), and the mobile phase B comprised 0.1% FA and 80% ACN. The liquid phase gradient settings are as follows: 0 min to 5 min: 4% to 10% mobile phase B; 5 min to 85 min: 10% to 22% mobile phase B; 85 min to 110 min: 22% to 40% mobile phase B; 110 min to 111 min: 40% to 95% mobile phase B; and 110 min to 111 min: 95% mobile phase B. The flow rate was maintained at 300 nL/min [19]. The separated peptide was introduced directly into the Orbitrap Fusion Lumos Tribrid Mass Spectrometer for online detection. The raw MaxQuant (1.6.2.10) mass spectrum files were searched against the UniProt database.

### Intrathecal and Intraspinal Injection

#### Intrathecal Injection

Saikosaponin C (SSc), the active ingredient in Bupleurum, inhibits abnormal Tau phosphorylation [20]. The intrathecal injection was performed as described by Li *et al.* [21]. In short, mice were anesthetized with 5% isoflurane and anesthesia was maintained with 3% isoflurane during surgery. The mouse pelvic girdle was firmly fixed on a 15-mL centrifuge tube to fully expose the L5–6 intervertebral space. A 30 G needle attached to a 25-µL Hamilton syringe was inserted into the L5–6 intervertebral space. A slight tail flick confirmed entry into the subarachnoid space. Approximately 5 µL of SSc (36 mmol/L; MedChemExpres, Shanghai, China) or the adeno-associated virus carrying the phosphorylation-mimicking variant GSK3B^Y216D^, AAV9-CAG-p-*Gsk3b* -EGFP (WZ Biosciences Inc. Jinan, China), and sterile saline (0.9% NaCl), or the control virus AAV9-CAG-EGFP (WZ Biosciences Inc., Jinan, China) was injected slowly, and the needle was left for 2 min before withdrawal. To ensure the infection rate of the virus, injections were administered daily for three consecutive days.

#### Intraspinal Injection

The mice were anesthetized with sodium pentobarbital (1%, 100 mg/kg; IP), and erythromycin eye ointment was applied to each eye. After disinfection with iodophor, in the prone position, the back skin was cut open at the highest point of the dorsal eminences to expose the vertebrae. The spine was fixed on a stereotactic frame (RWD Life Science Inc., Shenzhen, China), and the muscles and dura covering the intervertebral space were removed with forceps to reveal the white spinal cord. About 500 nL of pAV-U6-RFP-*Mapt*-shRNA or scrambled adeno-associated virus pAV-U6-RFP-shRNA (WZ Biosciences Inc. Jinan, China), and 500 nL of pAV-CMV-*Mapt*-RFP or scrambled adeno-associated virus pAV-CMV- RFP (WZ Biosciences Inc. Jinan, China) were injected into the lumbar spinal cord with a glass micropipette. The insertion depth of the needle tip was 400 μm below the surface of the spinal cord and the speed was 50 nL/min. After the injection, the needle was left for 5 min before removal.

### Behaviors

#### Mechanical Pain

Following the method described by Chaplan *et al.* [22], the 50% mechanical pain threshold was quantified by measuring the withdrawal response of the left hind paw to von Frey filament stimulation. The mice were acclimated to the observation chambers for ~1 h before the test. The method followed the up-down principle: “X” was recorded when the paw responded to ciliary stimulation, and “O” was recorded when there was no response. In the absence of a response, the filament of the next greater force was applied. Following a response, the filament of the next lower force was applied. The interval between each test was set to at least 1 min. The 50% g threshold = (10 ^(xf+kδ)^)/10000, where xf = value (in log units) of the final von Frey hair used; k = tabular value for the pattern of positive/negative responses; and δ = mean difference (in log units) between stimuli (here 0.296).

#### Thermal Pain

The mice were placed in test chambers on a glass plate in advance until they were quiet. The light emitted by the analgesic heating source passed through the glass plate to the paw surface. When the stimulated paw withdrew, the light turned off. To eliminate tissue damage, the stimulation disconnected automatically after 20 s. The test was conducted three times at 5-min intervals. The average of three paw withdrawal latencies was considered to be the thermal pain threshold.

#### Open Field

The open-filed chamber was a white plastic box (dimensions: 45 cm × 45 cm × 45 cm). The mice were placed in one corner of the chamber and allowed to freely explore for 5 min. The chamber was wiped with 75% alcohol after each test. The movement was recorded using ANY-maze software (Stoelting, Wood Dale, IL, USA). The total distance moved and time spent in the center were evaluated.

#### Elevated Plus Maze

The mice were adapted for 1 h in the behavioral testing room before the test. They were gently placed in the center of a cross with two open arms and two closed arms, with their heads facing the open arms. They were allowed to freely explore for 5 min. The elevated cross was wiped with 75% alcohol after each test. The movement was recorded using ANY-maze software. Time in the open arm and entries into the open arm were recorded.

### Immunofluorescence and Confocal Imaging

The mice were anesthetized with sodium pentobarbital (1%, 100 mg/kg; IP) and perfused transcardially first with phosphate-buffered saline (PBS) until all blood was washed out and second with 4% paraformaldehyde (PFA) for fixation. The L4–6 region of the spinal cord was dissected and immersed in 4% PFA overnight at 4 °C. The region was then placed in 15% sucrose for 1 day, followed by 30% sucrose for at least 2 more days. The region was cut into 25-μm-thick sections on a cryostat (Thermo Fisher Scientific). The sections were blocked in PBS containing 0.3% TritonX-100 and 5% normal donkey serum for 1 h at room temperature and stained with the primary antibodies, Tau (mouse, 1:200, Biofarm, OB-MMS033-01, Hangzhou, China), Tau (rabbit, 1:200, Biofarm, OB-PRB108-01), FITC-conjugated isolectin B4 (FITC-IB4, mouse, 1:200, Sigma, L2895), calcitonin gene-related peptide (CGRP, mouse, 1:200, Abcam, ab81887, Cambridge, MA, USA), MAP2 (rabbit, 1:200, Cell Signaling Technology, 4542S, Danvers, Massachusetts, USA), NeuN (mouse, 1:500, Abcam, ab104224), c-Fos (rabbit, 1:1000, Cell Signaling Technology, 2250), Iba1 (goat, 1:200, Abcam, ab5076), Iba1 (rabbit, 1:200, Cell Signaling Technology, 17198), GFAP (mouse, 1:300, Cell Signaling Technology, 3670), GFAP (rabbit, 1:200, Cell Signaling Technology, 80788S), IL-1β (rabbit, 1:200, Abcam, ab9722), and BDNF (rabbit, 1:200, Abcam, ab108319) at 4 °C overnight. After three rinses with TPBS, the sections were incubated with the Alexa Fluor™ 488 donkey anti-mouse secondary antibody (Abcam, ab150105), Alexa Fluor™ 594 donkey anti-rabbit secondary (Abcam, ab150108), Alexa Fluor™ 488 donkey anti-goat secondary antibody (Thermo Fisher Scientific, A-11055, WA, USA), Cyanine5 goat anti-rabbit IgG secondary antibody (Thermo Fisher Scientific, A10523), and Alexa Fluor™ 594 goat anti-mouse IgG secondary antibody (Thermo Fisher Scientific, A-11005) for 1 h at room temperature. After three gentle washes of 5 min with TPBS, the sections were mounted using Prolong gold antifade reagent with DAPI (Invitrogen, P36931, Carlsbad, CA, USA). Images were captured using confocal (Nikon A1R; Nikon, Japan) or standard (x-cite 120; Olympus, Japan) fluorescence microscopy. ImageJ software was used for statistical analysis.

### Western Blot

The mice were sacrificed immediately after isoflurane anesthesia, and the L4–6 spinal cord was harvested. The extracted tissue was homogenized in lysis buffer containing protease and phosphoprotease inhibitors and centrifuged (12,000 r/min, 15 min, at 4 °C), and the supernatant was collected as the protein sample. Protein concentrations were determined using a BCA Protein Assay Kit (Beyotime, P0009, Nantong, China). The protein sample was combined with 5× SDS and lysis buffer and boiled at 95 °C for 8 min. Proteins were isolated using SDS/PAGE and transferred to a PVDF membrane. The PVDF membranes were blocked with 5% nonfat milk (for nonphosphorylated protein) or 5% BSA (for phosphorylated protein) in TPBS for 1 h, then incubated with the primary antibodies, Tau (rabbit, 1:1000, Cell Signaling Technology, 46687), p-Tau (T231) (mouse, 1:200, Thermo Fisher Scientific, AT180), IL-1β (rabbit, 1:1000, Abclonal, A16288, Wuhan, China), BDNF (rabbit, 1:1000, Abcam, ab108319), GAPDH (rabbit, 1:5000, Diagbio, db106), Actin (rabbit, 1:5000, Abclonal, AC026), GSK3B (mouse, 1:1000, Cell Signaling Technology, 9832), p-GSK3B (Y216) (rabbit, 1:1000, Thermo Fisher Scientific, 44-604G), p-GSK3B (Ser9) (rabbit, 1:1000, Cell Signaling Technology, 14630), TNFa (rabbit, 1:1000, Abclonal, A11534), IL6 (rabbit, 1:1000, Abclonal, A2447), IL18 (rabbit, 1:1000, Abcam, ab191860), GFP (rabbit, 1:1000, Abcam, ab290), and Flag (rabbit, 1:1000, Abclonal, AE092), at 4 °C overnight. The blots were then incubated with HRP-conjugated goat anti-mouse or goat anti-rabbit secondary antibodies for 1 h at room temperature. The membranes were imaged using the ChemiDoc Touch Imaging System (BioRad, USA). Images were quantified using Gelpro32.

### Statistical Analyses

We present the western blot and immunofluorescence data as the mean ± standard deviation (SD). The data from behavior tests, including mechanical pain, thermal pain, open field, and elevated plus maze, are expressed as the mean ± standard errors of the means (SEM). Comparations between the two groups were performed using a two-tailed, unpaired *t-*test. One-way analysis of variance (ANOVA) with Tukey's multiple comparison tests was used to determine differences between the three groups. For behavioral tests for mechanical pain and thermal pain, two-way ANOVA with repeated measures following Sidak's multiple comparisons tests was used to compare differences between two groups at different time points, and two-way ANOVA with repeated measures following Tukey's multiple comparisons tests was used to compare differences among three groups at distinct time points. Statistical comparisons were performed with GraphPad Prism 8. Probability (*P*) values <0.05 were considered statistically significant.

## Results

### Tau Hyperphosphorylated in Spinal L4–6 Under CFA-induced Chronic Inflammatory Pain Conditions

**Fig. 1:**
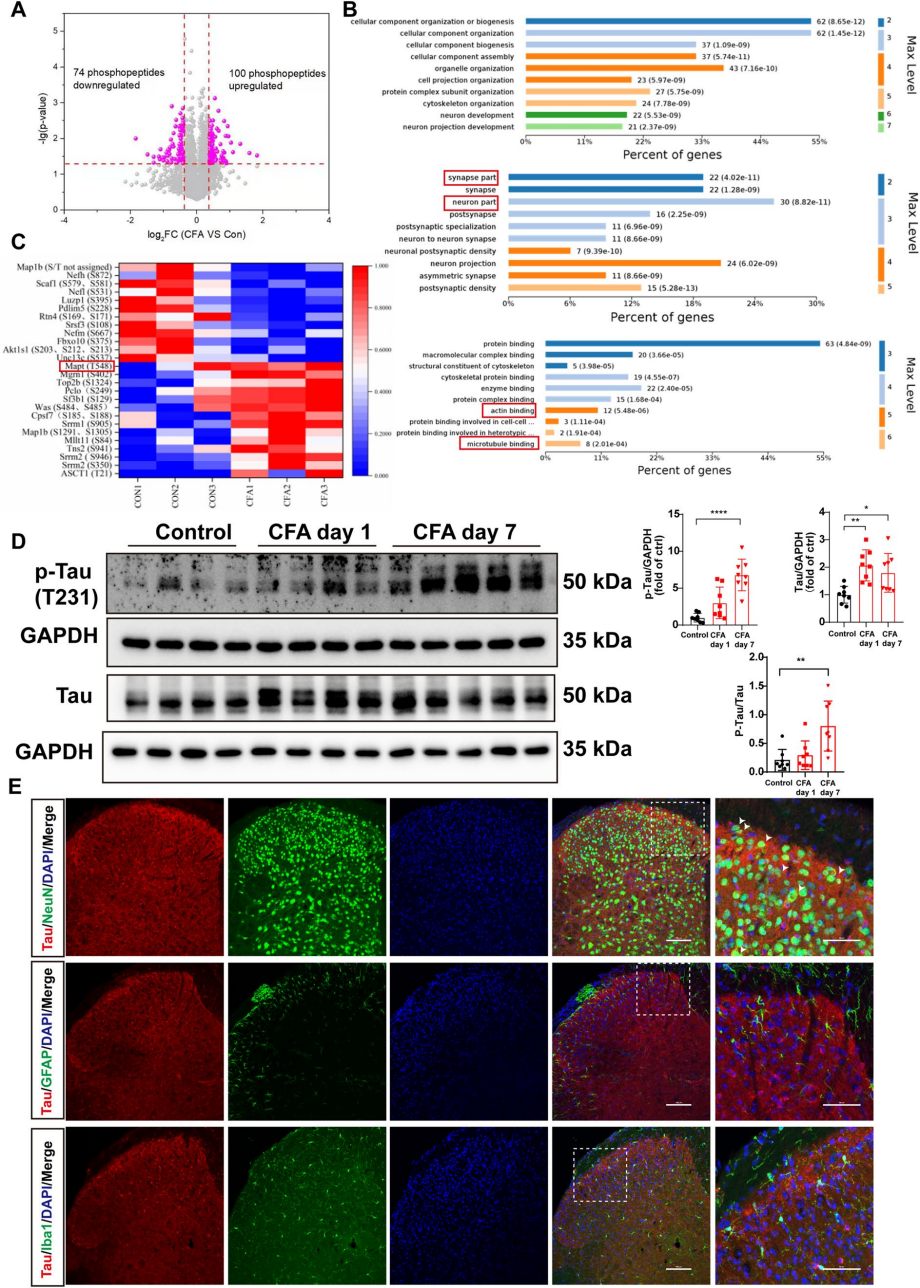
Tau is hyperphosphorylated in spinal L4–6 under CFA-induced chronic inflammatory pain conditions**. A** Volcano plot of differentially-expressed phosphopeptides. **B** GO function of upregulated/downregulated DEGs including biological process, cellular component, and molecular function. The images show the 10 most prominent GO nodes. **C** Heat map of selected differentially-expressed phosphopeptides; **D** Protein expression of p-Tau (T231) and total Tau in the spinal L4-6 as assessed in the CFA-treated group *versus* the sham group. Western blot analysis normalized to GAPDH (*n* = 8 from 5 mice per group, *P <0.05, **P <0.01, ****P <0.0001, one-way ANOVA). **E** Tau (red) immunostained with Neuronal Nuclei (NeuN; a neuronal marker, green; left), glial fibrillary acidic protein (GFAP; an astrocyte marker, green; middle), and ionized calcium binding adaptor molecule-1 (Iba-1; a microglia marker, green; right). Blue, DAPI staining. White arrowheads indicate the colocalization of Tau (red) and cell markers (green). Scale bars, 50 μm and 100 μm.

According to a previous study, CFA successfully causes inflammatory pain, as manifested by a decrease in the threshold for mechanical pain and thermal pain after CFA injection (Fig. S1A). A significant increase of c-Fos-positive neurons in the L4–6 spinal dorsal horn was observed on days 1 and 7 after CFA injection (Fig. S1B), indicating that CFA-induced pain was indeed transmitted through the spinal dorsal horns of L4–6. Notably, the 7-day inflammatory pain did not affect the mobility and anxiety-like behaviors (Fig. S1C, D). We then used quantitative *TMT* mass spectrometric sequencing to enrich phosphorylated peptide in the lumbar 4–6 of rats after CFA or saline injection. Approximately 16,700 peptides and 10,696 phosphorylated peptides were identified. We found 74 significantly downregulated phosphorylated peptides and 100 upregulated phosphorylated peptides through phosphorylation omics analysis of rat spinal L4–6 (Fig. 1A). Differentially-phosphorylated peptides corresponded to 71 upregulated and 55 downregulated genes (Fig. S2A). We compiled some differential phosphorylated peptides into heat maps (Fig. 1C). Compared with the saline injection group, the phosphorylation of microtubule-associated protein tau at Thr 548 was increased in the CFA 7-day group. The Thr548 site in rats corresponds to the mouse Thr231. GO function analysis, including biological process (BP), cellular component (CC), and molecular function (MF) was applied to the differential genes (DEGs). The top 10 BP, CC, and MF are shown in Fig. 1B. In the CC category, the significantly enriched terms included “synapse part” and “neuron part”. MFs mainly involved protein binding, such as microtubule binding. Notably, Tau is a microtubule-binding protein and is considered a neural marker. To verify whether Tau expression was increased after CFA injection, we assessed the protein expression of Tau on days 1 and 7 after CFA through western blotting. Consistently, total Tau was increased on days 1 and 7 after CFA, and Tau phosphorylation on Thr231 in the CFA group was significantly higher on day 7 than that in the saline group (Fig. 1D). Furthermore, we discovered a significant colocalization of Tau with CGRP- and IB4-labeled axon terminals in lamina I and outer lamina II (IIo) of the dorsal cord (Fig. S3A). This colocalization was also observed with another neuronal marker, microtubule-associated protein 2 (MAP2) in the dorsal cord (Fig. S3B). On day 7 after CFA injection, Tau was indeed expressed in neurons, but not in astrocytes and microglia (Fig. 1E). These results suggest that Tau is hyperphosphorylated and accumulates in neurons of L4–6 after CFA treatment.

### IL-1β and BDNF Levels are Increased After CFA Injection

**Fig. 2:**
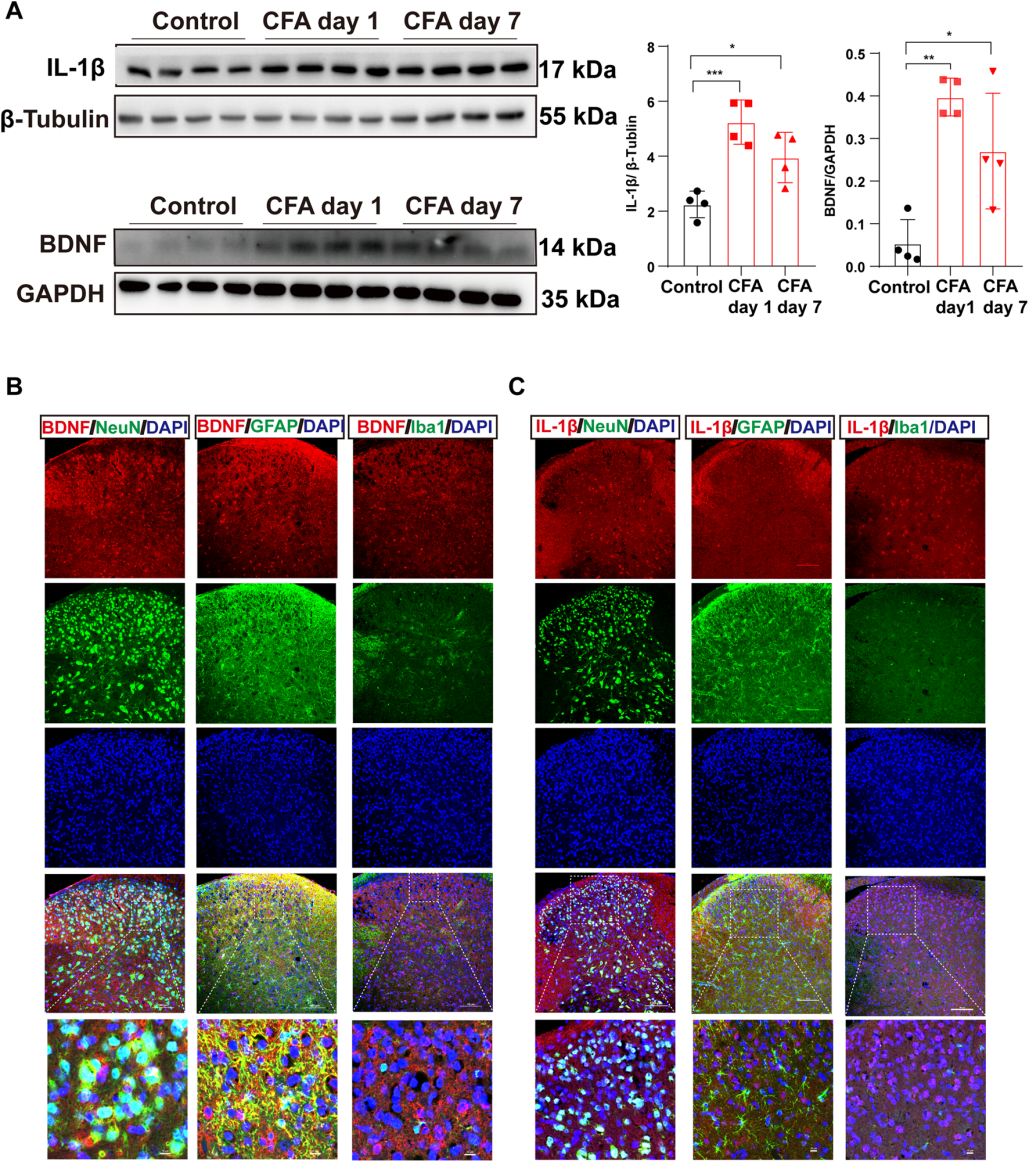
IL-1β and BDNF levels increase after CFA injection. **A** Western blots and analysis of protein levels of IL-1β and BDNF of L4–6 in the control group and on days 1 and 7 after CFA injection (*n* = 4 per group, *P <0.05, **P <0.01, ***P <0.001, one-way ANOVA followed by Tukey's multiple comparisons test). **B** Images showing the subcellular colocalization of BDNF in the spinal dorsal cord 7 days after CFA injection. Immunofluorescence double stain shows that BDNF (red) mainly co-stains with neurons (NeuN, green) and astrocytes (GFAP, green), rarely with microglia (Iba1, green). **C** Images showing the subcellular colocalization of IL-1β in the spinal dorsal cord 7 days after CFA injection. Double immunofluorescence staining reveals that IL-1β (red) primarily co-stains with neurons (NeuN, green) across the spinal dorsal cord and astrocytes of layer I of the dorsal cord (GFAP, green), but rarely with microglia (Iba1, green). Scale bars, 10 μm and 100 μm.

We then assessed the expression change and examined the cell-type localization of the inflammatory cytokines IL-1β and BDNF in the spinal cord after CFA injection because of the evidence of their pro-nociceptive role [23, 24]. The western blot analysis revealed a clear increase of IL-1β and BDNF on days 1 and 7 after CFA (Fig. 2A). Double staining revealed that BDNF was expressed in neurons (NeuN) and astrocytes (GFAP), but rarely in microglia (Iba1) in the spinal dorsal cord on day 7 after CFA injection (Fig. 2B). On day 7 post-CFA injection, IL-1β was predominantly expressed in neurons across the dorsal cord, and to a lesser extent in astrocytes within the superficial layers of the dorsal horn (Fig. 2C).

### Tau is Required for Pain Hypersensitivity in CFA-induced Inflammatory Pain

**Fig. 3:**
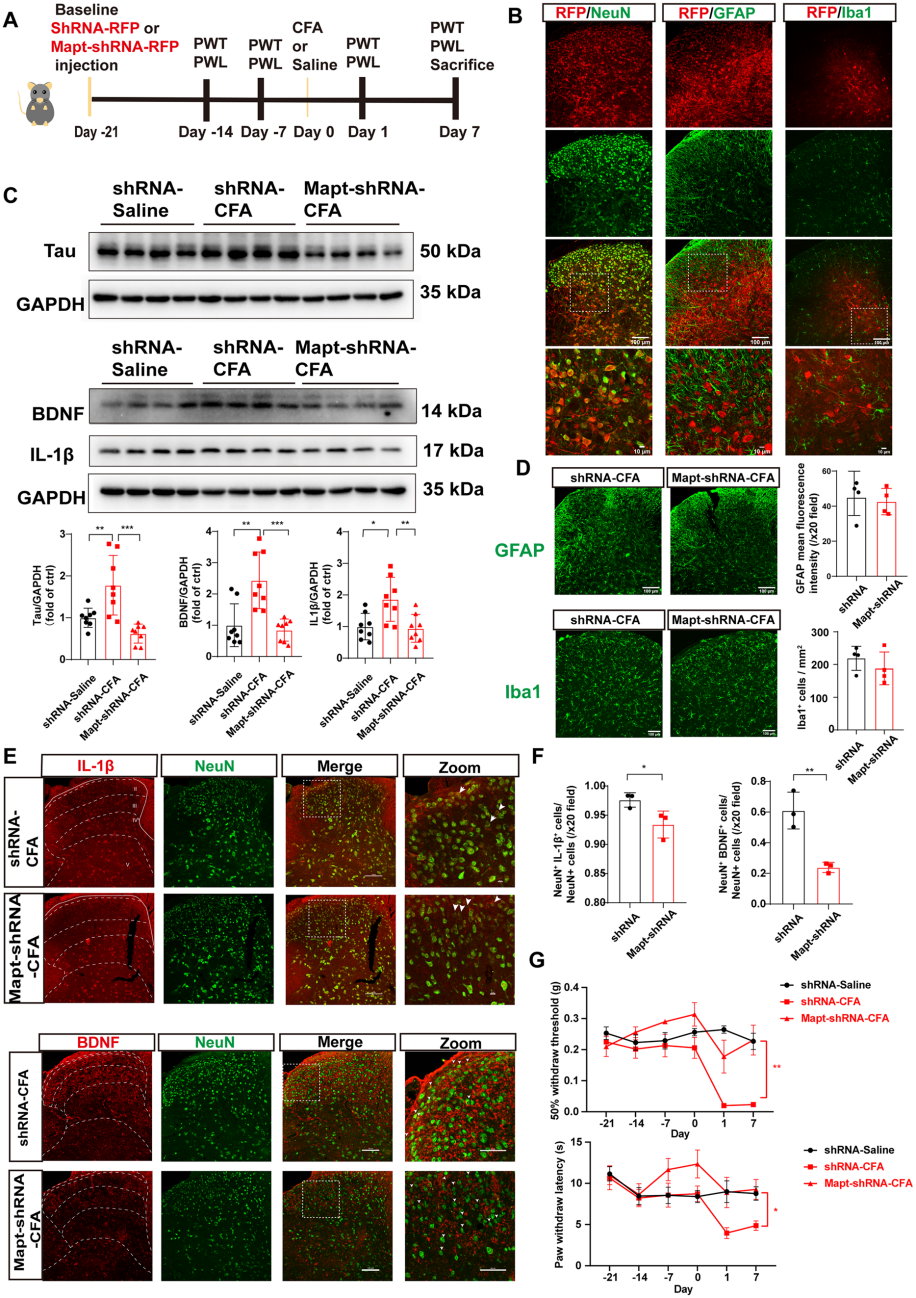
Tau is required for pain hypersensitivity in CFA-induced inflammatory pain. **A** Timeline schematic of the experimental paradigm. **B** Double immunofluorescence colocation shows that Mapt-shRNA (red) is predominantly expressed in neurons (NeuN, green), but not in astrocytes (GFAP, green) and microglia (Iba1, green). **C** Tau, IL-1β and BDNF protein levels of L4–6 in the shRNA-saline, shRNA-CFA, and Mapt-shRNA-CFA groups (*n* = 8 per group, *P <0.05, **P <0.01, ***P <0.001, one-way ANOVA with Tukey's multiple comparisons test). **D** Immunofluorescence staining of GFAP (upper) and Iba1 (lower) in the dorsal L4–6 between the shRNA-CFA group and the Mapt-shRNA-CFA group. Blue, DAPI staining (*n* = 4 per group, unpaired *t*-test). **E** Upper images show the colocalization of IL-1β (red) and NeuN (green) in the dorsal cord in the shRNA-CFA group and the Mapt-shRNA-CFA group. White arrowheads in the Mapt-shRNA-CFA group indicate that IL-1β (red) expression is not found in the neurons (green). Lower images show the colocalization of BDNF (red) and NeuN (green) in the dorsal cord in the shRNA-CFA group and the Mapt-shRNA-CFA group. White arrowheads in the Mapt-shRNA-CFA group indicate that BDNF (red) expression is not found in the neurons (green). **F** Statistical results of the E plot (*n* = 3 per group, *P <0.05, unpaired *t-*test). **G** Mechanical and thermal pain measured before virus injection (day -21), CFA injection (day 0), and days 1 and 7 after CFA injection. (*n* = 7 each for the shRNA-saline and shRNA-CFA groups and* n* = 8 for the Mapt-shRNA-CFA group, *P <0.05, **P <0.01, two-way ANOVA with repeated measures followed by Tukey’s multiple comparisons tests). Scale bars, 10 μm, 50 μm, and 100 μm.

To determine whether Tau downregulation in L4–6 alleviated CFA-induced pain hypersensitivity through upregulation of IL-1β and BDNF, we used an adeno-associated virus carrying *Mapt*-shRNA to specifically knock down Tau in L4–6 (Fig. 3A). Scrambled shRNA was used as a negative control. Assays for Tau protein alteration and red fluorescent protein (RFP) fluorescence were applied to determine the effectiveness of viral knockdown. The western blot results revealed that *Mapt*-shRNA significantly down-regulated the increased Tau induced by CFA (Fig. 3C). Strong RFP fluorescence was detected in spinal L4–6 (Fig. 3B). Moreover, the double fluorescent co-localization assay of RFP with neurons (NeuN), astrocytes (GFAP), and microglia (Iba1) revealed that the virus we constructed was predominantly expressed in neurons (Fig. 3B). To study the influence of Tau on IL-1β and BDNF, we used western blotting to detect the expression of these two factors after *Mapt*-shRNA treatment. The results demonstrated that the protein expression of IL-1β and BDNF in the CFA + *Mapt*-shRNA group was lower than that in the CFA + shRNA (scrambled) group (Fig. 3C). Immunofluorescence staining of the astrocyte marker (GFAP) and microglia marker (Iba1) revealed that the injection of *Mapt*-shRNA after CFA did not activate or inhibit these two types of glia (Fig. 3D). Because Tau is basically expressed in neurons, and IL-1β and BDNF in the dorsal horn are largely expressed in neurons, we used immunofluorescence co-localization to explore whether Tau knockdown in neurons after CFA would reduce IL-1β and BDNF accumulation in neurons. Double immunofluorescence staining revealed that spinal injection of *Mapt* - shRNA attenuated the CFA-induced production of IL-1β and BDNF in neurons of the dorsal cord (Fig. 3E). The statistical results are shown in Fig. 3F. Consistently, behavioral tests of mechanical and thermal pain demonstrated that knocking down Tau in the spinal cord significantly alleviated CFA-induced pain on day 7 (Fig. 3G). Collectively, these findings suggest that Tau is required for pain hypersensitivity in CFA-induced inflammatory pain, and *Mapt*-shRNA reduced the elevated IL-1β and BDNF expression induced by CFA.

### Pharmacological Inhibition of Tau Phosphorylation Relieves CFA-induced Pain

**Fig. 4:**
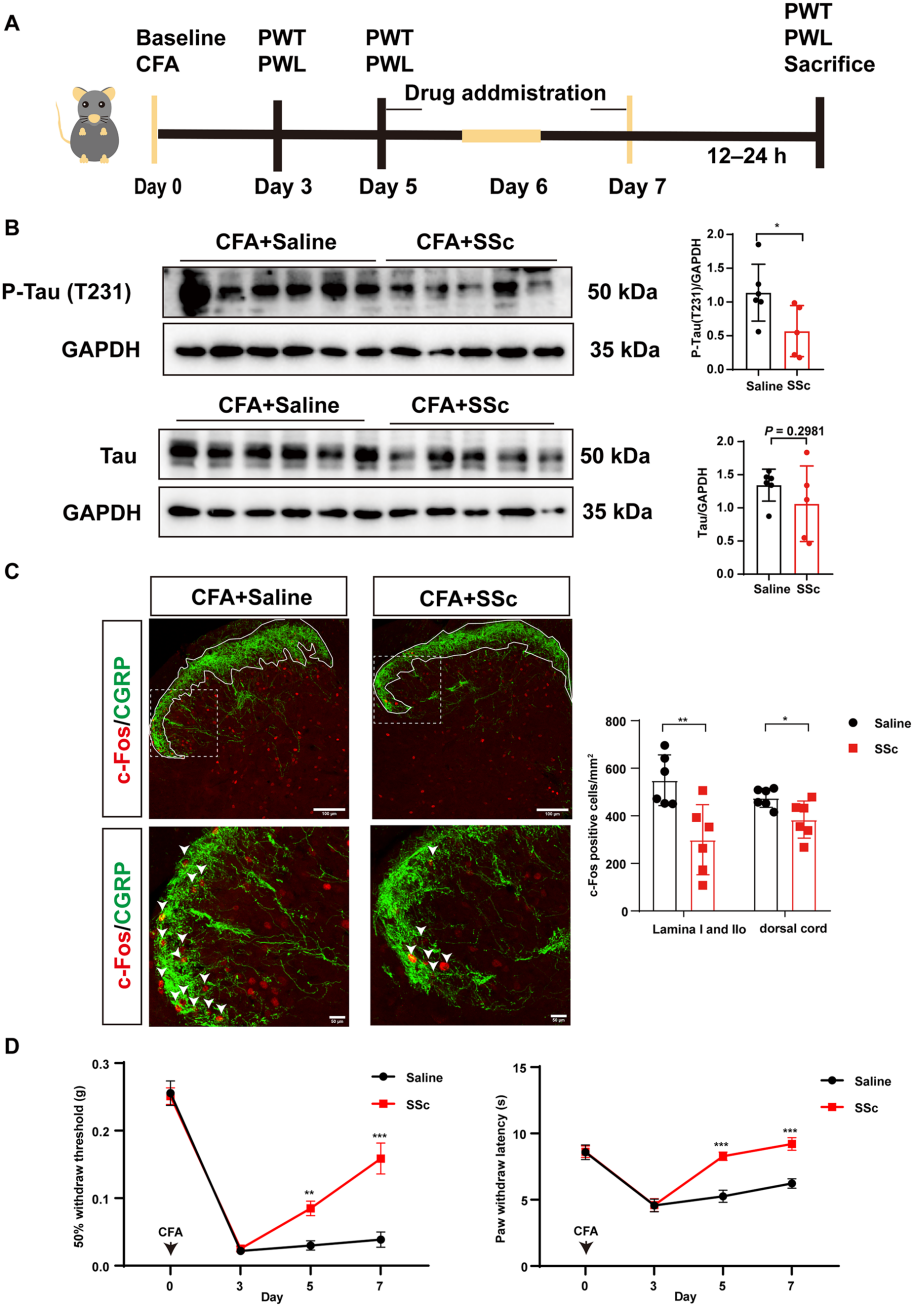
Pharmacological inhibition of tau phosphorylation relieves CFA-induced pain. **A** Timeline of the experimental paradigm. **B** Western blots and analysis of protein expression of p-Tau (T231) and total Tau in L4–6 in the CFA + saline and CFA+ SSc groups, normalized to GAPDH (*n* = 6 for CFA + saline group, *n* = 5 for CFA + SSc group, *P <0.05, unpaired *t*-test,). **C** Representative immunofluorescence images showing the colocalization of c-Fos and CGRP in the dorsal cord in the CFA + Saline and CFA+ SSc groups (*n* = 6 per group, *P <0.05, **P <0.01, unpaired *t*-test). **D** Mechanical and thermal pain before (day 0), after CFA injection (day 3), and during SSc injection (days 5 and 7) (mechanical pain, *n* = 12 per group; thermal pain, *n* = 12 for saline group and *n* = 10 for SSc group, **P <0.01, ***P <0.001, two-way ANOVA with repeated measures followed by Sidak’s multiple comparisons tests). Scale bars, 50 μm and 100 μm.

To determine whether inhibiting hyperphosphorylated Tau (Thr231) could relieve pain, we injected SSc intrathecally into mice on days 5 to 7 after CFA injection. SSc is a drug thought to inhibit Tau phosphorylation at Thr 231 [20]. A flow chart is shown in Fig. 4A. The western blot results revealed that the hyperphosphorylation of p-Tau (Thr231) was markedly suppressed after intrathecal injection of SSc (Fig. 4B). The protein level of total Tau did not change (Fig. 4B). In addition, the results from western blot analysis indicated that SSc administration reduced the protein expression of IL-1β and BDNF (Fig. S5A). To better define the extent of pain signaling in the crucial regions of laminae I and IIo of the dorsal horn, we co-labelled c-Fos with CGRP and IB4. The immunofluorescence staining targeting c-Fos in both laminae I and IIo as well as across the entire dorsal horn layer demonstrated notably fewer c-Fos-positive cells in the SSc group than in the saline group (Figs. 4C and S5B). These results suggested that SSc restrained the increased neuronal activation induced by CFA. Moreover, pain-related behaviors indicated that intrathecal injection of SSc dramatically attenuated the CFA-induced pain hypersensitization (Fig. 4D).

### Tau Overexpression Induces Hyperalgesia Through Increased Levels of IL-1β and BDNF

**Fig. 5:**
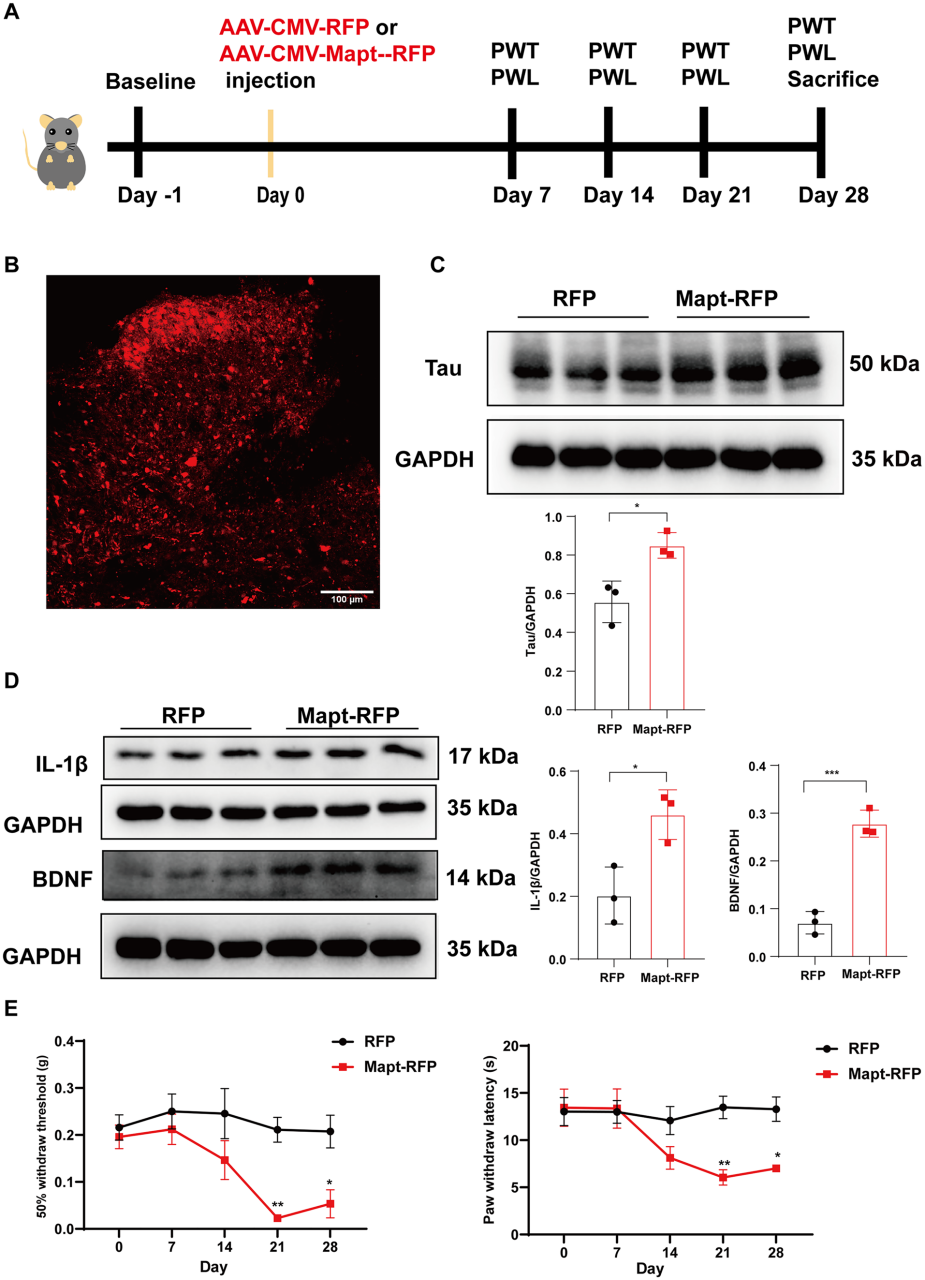
Tau overexpression induces hyperalgesia through increased levels of IL-1β and BDNF. **A** Timeline of the experimental paradigm. **B** Fluorescence image of RFP in the dorsal cord after AAV-CMV-Mapt-RFP virus injection. **C** Western blots and analysis of the protein expression of Tau in L4–6 in the RFP and Mapt-RFP groups, normalized to GAPDH (*n* = 3 per group, *P <0.05, unpaired *t*-test. **D** Western blots and analysis of the protein expression of IL-1β and BDNF in L4–6 in the RFP and Mapt-RFP groups normalized to GAPDH (*n* = 3 per group, *P <0.05, ***P <0.001, unpaired *t*-test). **E** Mechanical and thermal pain measured before virus injection (day 0) and on days 7, 14, 21, and 28 after the injection (*n* = 7 per group, *P <0.05, **P <0.01, two-way ANOVA with repeated measures followed by Sidak’s multiple comparisons tests). Scale bar, 100 μm.

To further test whether Tau accumulation is sufficient to induce hyperalgesia, wild-type mice were injected with adeno-associated virus overexpressing Tau into the spinal dorsal cord. The experimental scheme of intraspinal administration of the Tau overexpression virus is shown in Fig. 5A. The strong red fluorescence (Fig. 5B) and high expression of the fusion protein Flag (Fig. S6A) suggested that the virus was expressed in L4–6 of the spinal cord. Western blotting was applied to examine the overexpression efficiency of virus infection. The results revealed that the protein expression of Tau was increased after infection with the virus carrying the *Mapt* gene (Fig. 5C). We further evaluated IL-1β and BDNF expressions in the spinal cord after Tau overexpression. The western blotting results revealed that Tau overexpression robustly upregulated the levels of IL-1β and BDNF (Fig. 5D). Notably, the pain-related behavior results implied that Tau overexpression induced hyperalgesia (Fig. 5E).

### Hyperphosphorylation of GSK3B (Tyr216) Induces Tau Accumulation in CFA-Induced Pain

**Fig. 6:**
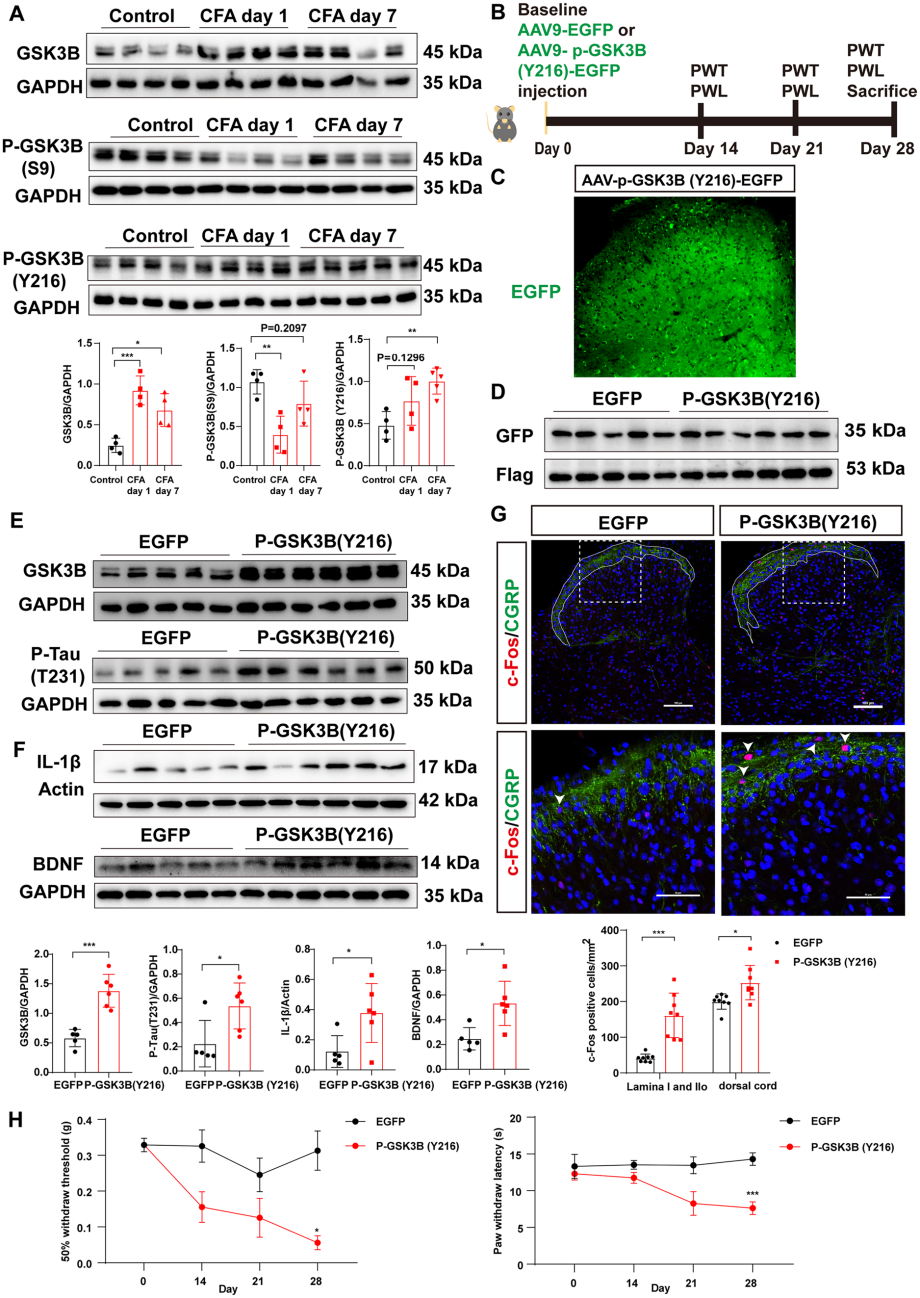
Hyperphosphorylation of GSK3B (Tyr216) induces Tau accumulation in CFA-induced pain. A Western blots and analysis of the protein levels of GSK3B, p- GSK3B (Ser9), and p- GSK3B (Tyr216) of L4–6 in the control group and on days 1 and 7 after CFA injection normalized to GAPDH (*n* = 4 per group, *P <0.05, **P <0.01, ***P <0.001, one-way ANOVA followed by Tukey's multiple comparisons tests). **B** Timeline of the experimental paradigm. **C** Fluorescence image of EGFP in the dorsal cord after AAV-CMV-p-GSK3B(Y216)-EGFP virus injection. **D** Western blots of protein expression of the non-fusion protein GFP and the fusion protein Flag in the EGFP group and the p-GSK3B (Y216)-EGFP group. **E** Western blots and analysis of protein levels of GSK3B and p-Tau (T231) of L4-6 in the EGFP and p-GSK3B (Y216)-EGFP groups normalized to GAPDH (*n* = 5 for the EGFP group and *n* = 6 for the p-GSK3B (Y216)-EGFP group, *P <0.05, ***P <0.001, unpaired *t*-test). **F** Western blots and analysis of the protein levels of IL-1β and BDNF of L4–6 in the EGFP and p-GSK3B (Y216)-EGFP groups (IL-1β is normalized to Actin and BDNF is normalized to GAPDH) (*n* = 5 for the EGFP group and *n* = 6 for the p-GSK3B (Y216)-EGFP group, *P <0.05, unpaired *t*-test). **G** Representative immunofluorescence images showing the colocalization of c-Fos and CGRP in the dorsal cord in the EGFP and p-GSK3B(Y216)-EGFP groups (*n* = 8 from 4 mice per group, *P <0.05, ***P <0.001, unpaired *t*-test, 2-tailed). **H** Mechanical and thermal pain before virus injection (day 0) and on days 14, 21, and 28 after the injection (*n* = 6 per group, *P <0.05, ***P <0.001, two-way ANOVA with repeated measures followed by Sidak's multiple comparisons tests). Scale bars, 50 μm and 100 μm.

GSK-3B is a common serine/threonine kinase of Tau, so we suspected that the excessive accumulation of Tau might be mediated by GSK3B. First, we determined the protein expression level of total GSK3B after CFA modeling through western blotting. The results manifested that the expression of GSK3B was upregulated on days 1 and 7 after CFA injection (Fig. 6A). The phosphorylation of GSK3B at Ser9 deactivates GSK3B, whereas its phosphorylation at Tyr216 activates it [25, 26]. The western blot results also revealed that the phosphorylation of Ser9 of GSK3B in the CFA model group was decreased, whereas the phosphorylation of Tyr216 was increased compared with the saline group (Fig. 6A). To determine the causal relationship between GSK3B and Tau in the occurrence of pain, we constructed an adeno-associated virus carrying the phosphorylation-mimicking variant GSK3B^Y216D^, which simulates the phosphorylation of Tyr216 and GSK3B activation. The experimental scheme involved intrathecal administration of the virus and then observation of the pain behavior (Fig. 6B). Strong green fluorescence (Fig. 6C), and high expression of the non-fusion green fluorescent protein (GFP) and the fusion protein Flag (Fig. 6D) indicated that the virus was expressed in L4-6. The western blot results demonstrated that overexpression of the virus carrying the phosphorylation-mimicking variant GSK3B^Y216D^ was capable of increasing the protein expression of total GSK3B and p-Tau (T231) (Fig. 6E). Moreover, GSK3B activation promoted the production of IL-1β and BDNF (Fig. 6F). The increased number of c-Fos-positive cells in both lamina I and IIo, as well as throughout the entire dorsal horn layer in the p-GSK3B (Y216) group compared to the control group, provided evidence that GSK3B activation enhances neuronal excitability (Fig. 6G). As expected, GSK3B activation induced a significant reduction in paw withdrawal threshold and paw withdrawal latency (Fig. 6H). Taken together, these results indicated that GSK3B activation contributes to the hyperphosphorylation and accumulation of Tau.

## Discussion

Chronic inflammatory pain caused by tissue damage and persistent inflammation seriously affects the quality of a patient's life and sleep, and even brings more serious emotional disorders such as anxiety and depression. Unfortunately, current intervention measures for treatment are inadequate for pain relief, and nonsteroidal anti-inflammatory and opioid analgesic drugs cause multiple adverse effects. Hence, a better understanding of the mechanisms involved in chronic inflammatory pain is very important. Much attention has been focused on the contribution of Tau to AD, and little research has explored the role of Tau in emotions such as pain, anxiety, and depression. In the current study, we found that the expression of p-Tau (Thr231) was upregulated only on day 7 but not on day 1 after CFA. The protein expression of total Tau was increased on both CFA days 1 and 7 (Fig. 1D). For the first time, we demonstrated that overexpression of Tau in the spinal dorsal cord is sufficient and necessary to evoke nociceptive responses.

The microtubule-associated Tau protein is prominently localized in axons and neuron cell bodies [27]. Tau has multiple physiological functions such as regulating axonal transport and axonal elongation, modulating synaptic plasticity, and participating in neurogenesis [28]. Hugo et al. found that, compared with *Tau*^+/+^ mice, the response of *Tau* KO mice to acute noxious stimuli is decreased, whereas their pain-related behaviors are augmented under tonic inflammatory pain stimulation [9]. Later, they found that mice with spared nerve injury exhibit Tau hyperphosphorylation in the hippocampus owing to impaired autophagy, which in turn induces depressive-like behavior and cognitive impairment but not anxiety [10, 29]. In the von Frey test, Tau KO mice with spared nerve injury exhibit improved cognitive impairment but no pain relief. These studies suggested that the expression of Tau is altered after pain. However, these studies did not clearly explain the direct relationship between Tau hyperphosphorylation and pain, nor where these relationships occurred. Here, we first found that CFA-induced inflammatory pain triggered tau hyperphosphorylation and accumulation in the spinal L4-6 segments (Fig. 1). Remarkably, the *Mapt* - shRNA virus reversed CFA-induced pain behavior (Fig. 3G). Besides, reducing the phosphorylation at the threonine site 231 of Tau alleviated CFA-induced inflammatory pain (Fig. 4). Tau hyperphosphorylation leads to Tau aggregation and dysfunction, which is called tauopathy [30]. The imbalance between Tau kinase and phosphatase activities causes Tau hyperphosphorylation. The kinases that phosphorylate Tau comprise proline-directed protein kinases (PDPK), including GSK3B, cyclin-dependent kinase 5, and mitogen-activated protein kinases, non-PDPK, and tyrosine protein kinases [31]. GSK3B phosphorylation at Tyr216 is the active form. By contrast, phosphorylation at Ser9 is the inactive form. Previous studies have indicated that the phosphorylation at ser9 of GSK3B is decreased in mice with neuropathic pain *versus* untreated mice, and the inhibition of GSK3B activity attenuates neuropathic pain [32–34]. Here, we confirmed that the expression of GSK3B and GSK3B phosphorylation at Tyr216 (the active form) were up-regulated on days 1 and 7 after CFA injection (Fig. 6A). Moreover, we mutated the tyrosine at site 216 of GSK3B to aspartic acid (an acidic residue) to mimic persistent phosphorylation of Tyr216 and found that the GSK3B activation indeed promoted Tau accumulation in wild-type mice (Fig. 6E). Thus, the hyperphosphorylation and accumulation of Tau is partly mediated by GSK3B activation. Besides the abnormal activation of phosphokinases leading to Tau hyperphosphorylation, research has also shown that irregularities in the mTOR signaling pathway and autophagy regulation - as identified through KEGG enrichment analysis - can also contribute to abnormal Tau phosphorylation (Fig. S2B, C) [35, 36].

Accumulating evidence suggests that the central sensitization of the spinal cord contributes to the maintenance of chronic pain [37]. Inflammatory stimuli in rodent hind paws are sufficient to activate peripheral nociceptors and dorsal root ganglia, causing primary neurons to release various molecular mediators into the spinal dorsal cord including ATP, CCL2, TNFα, CSF-1, and BDNF [14]. These mediators activate microglia and astrocytes, leading to neuroinflammation, which drives the central sensitization of the spinal cord. Previous studies have found that the inflammatory factors TNFα and IL-6 in spinal L4–6 increase significantly on the third day after CFA treatment [38], whereas our results suggested that the inflammatory factors TNFα, IL-6, and IL18 did not increase significantly on days 1 and 7 after CFA injection (Fig. S4A-C). The expression levels of TNFα and IL6 may fluctuate with time [39]. IL-1β is widely acknowledged to significantly influence the development of CFA-induced inflammatory pain, whereas the role and regulation of IL18 in CFA-induced pain are less well understood [40, 41]. It is possible that IL18 is not significantly upregulated in this particular model, or the timing of its expression may be different from IL-1β, and its peak may occur at a different time point that was not evaluated in this study. Emerging research has demonstrated that increased TNFα and IL6 levels are predominantly localized in spinal and dorsal root ganglia neurons [38, 42]. Notably, our double immunofluorescence staining results indicated that IL-1β was also mainly localized in spinal neurons on post-injection day 7 (Fig. 2C), whereas previous studies have reported spinal astrocytes as a dominant source of IL-1β in the dorsal cord on day 3 post-CFA injection [43]. Such a discrepancy may be due to different pain states caused at distinct time points after CFA injection and mediated by diverse mechanisms. Notably, increasing numbers of studies have also reported caspase 1 activation, IL-1β cleavage, and the expression of inflammasome-forming NOD-like receptors in neurons [44–46]. Thus, the accumulation of Tau in neurons may directly activate caspase1 to increase mature IL-1β. Moreover, type 1 interleukin receptor (IL-1R1) is especially overexpressed within dorsal cord neurons but not glia in chronic inflammatory pain conditions [47]. Therefore, IL-1β in spinal neurons may directly bind to IL-1R1 to modulate synaptic transmission in spinal pain processing. Extensive evidence suggests that increased BDNF in spinal neurons regulates N-methyl-D-aspartate glutamate receptor-dependent synaptic plasticity, leading to central sensitization of the spinal cord [48, 49]. Consistently, we found that BDNF was significantly up-regulated on days 1 and 7 post-CFA injection. Central nervous system inflammatory responses may contribute to Tau aggregation [50, 51], whereas a pathological Tau load also triggers pro-inflammatory responses [52]. Although cell-to-cell transmission of pathological Tau activates glial cells, our results demonstrated that knocking down Tau in the spinal dorsal cord did not inhibit CFA-induced glial cell activation (Figs S4D–E, 3D) [53]. However, knocking down tau in neurons of the dorsal cord downregulated IL-1β and BDNF in spinal neurons (Fig. 3E). Notably, our results suggested that Tau accumulation upregulated mature BDNF appears contradictory to the reports which describe how hyperphosphorylation of Tau downregulates BDNF expression in AD [54, 55]. The following reasons may partly explain this inconsistency. First, the reported BDNF downregulation in the literature on Tauopathies and Tau transgenic mouse models is rather a direct consequence of amyloid pathology with the long-term accumulation of Tau [56]. However, the accumulation of Tau in our pain model was short-term, and only short-term viral intervention for Tau was given. Second, the increased BDNF promoted by Tau accumulation could represent a compensatory mechanism for neuronal impairment and synaptic deficits. Finally, Tau accumulation may activate the enzyme that cleaves pro-BDNF into mature BDNF [56].

In addition to neuroinflammation, synaptic loss and dysfunction are other major characteristics of Tauopathies. Pathogenic Tau in spinal neurons may directly cause synaptic plasticity changes and then cause pain [57]. Furthermore, the long-term accumulation of Tau in the central nervous system may explain why patients with chronic pain are more susceptible to suffering from AD [7, 8, 29]. Thus, the hyperphosphorylation of Tau in the spinal cord of AD patients may mediate their hyperalgesia clinically, based on the finding that over-expression of Tau in the spinal cord increases pain sensitivity. Additional studies are required to address these issues.

**Fig. 7:**
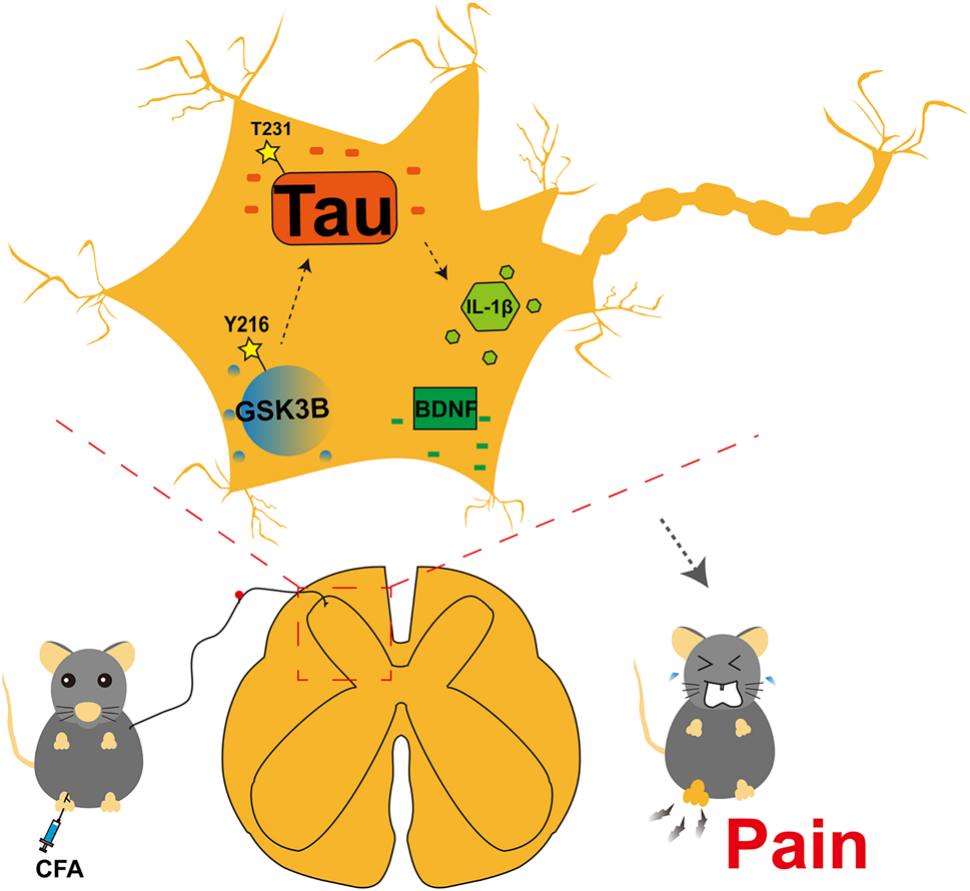
Schematic of Tau accumulation in the spinal cord contributing to chronic inflammatory pain through IL-1β and BDNF. GSK3B activation induces the hyperphosphorylation of Tau.

Taken together, the present study indicated that Tau accumulation in spinal neurons was a close participant in CFA-induced inflammatory pain through increased levels of IL-1β and BDNF in neurons (Fig. 7). These findings suggest that Tau in the dorsal horn could be a promising target for chronic inflammatory pain therapy.

### Supplementary Information

Below is the link to the electronic supplementary material.

Supplementary file1 (PDF 1709 kb)
