# Supplementary material for: Tau Accumulation in the Spinal Cord Contributes to Chronic Inflammatory Pain by Upregulation of IL-1β and BDNF
Source: Neurosci Bull. 2023 Dec 26;40(4):466–82. doi: 10.1007/s12264-023-01152-4 (PMC11003936; doi:10.1007/s12264-023-01152-4)
Supplement: Supplementary file 1 — Supplementary file1 (PDF 1709 kb) [file 12264_2023_1152_MOESM1_ESM.pdf]

Supplemental Figures and Figure Legends

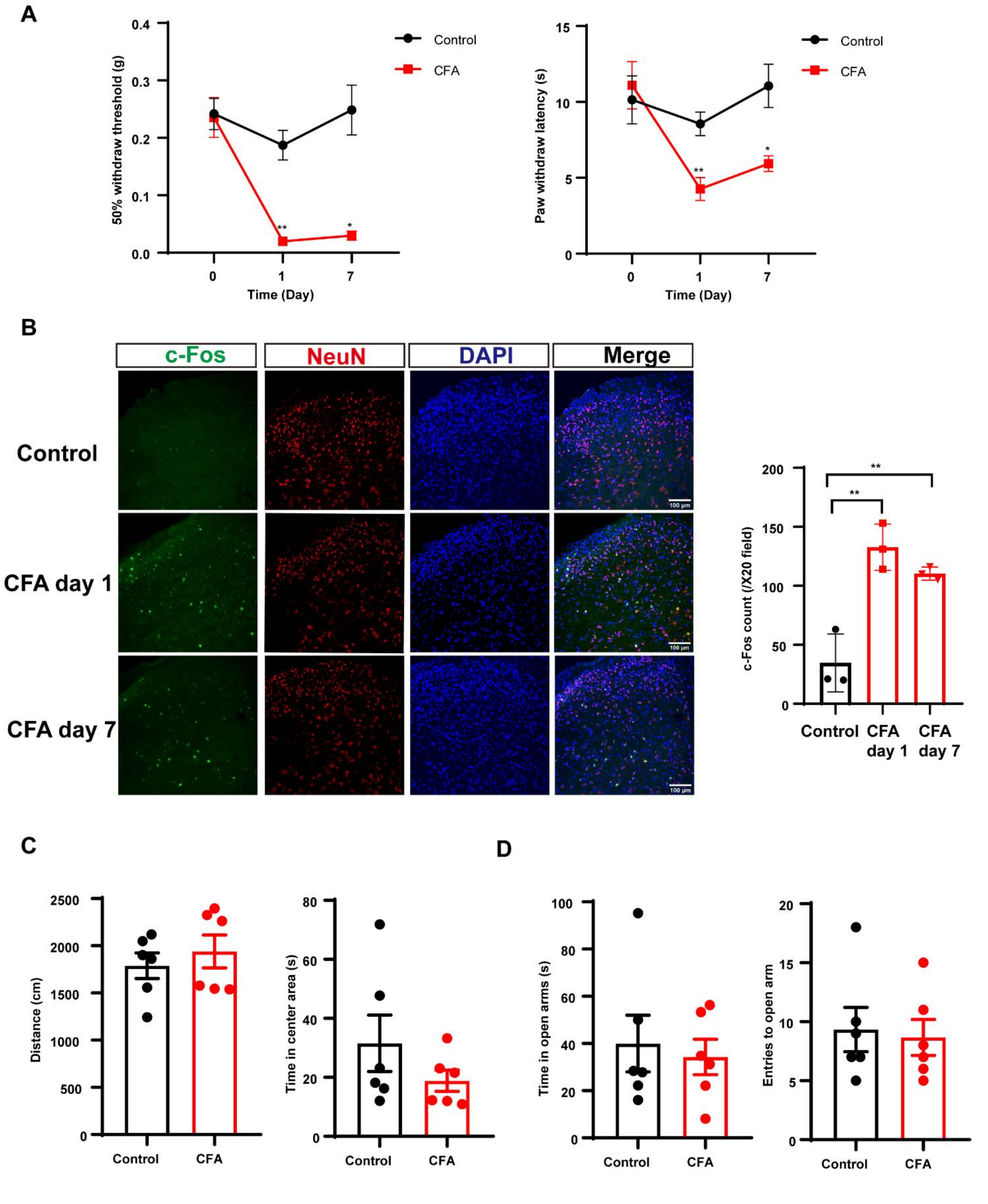

Fig. S1 Complete Freund's adjuvant (CFA)-induced inflammatory pain is transmitted into L4–6. **A** Mechanical and thermal pain before CFA injection (day0) and days 1 and 7 following CFA injection.

(n = 6 per group, *P <0.05, **P <0.01, two-way ANOVA with repeated measures followed by Sidak's multiple comparisons test). **B** c-Fos (green) coimmunostained with Neuronal Nuclei (NeuN; a neuronal marker, green). Blue, DAPI staining. (n = 3 per group, **P <0.01, unpaired t test). **C** Total distance moved and time spent in the center area in the OFT (n = 6 per group, unpaired t test). **D** Time in open arm and entries into open arm in the EPM (n = 6 per group, unpaired t test).

A

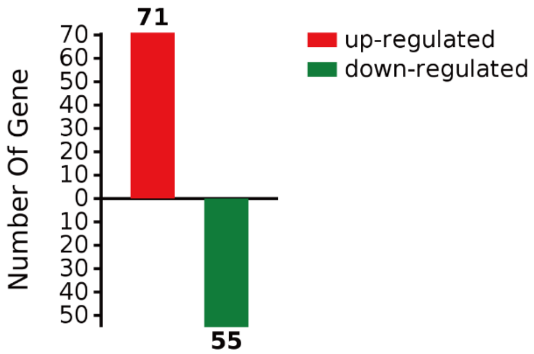
71
70
60
50
40
30
20
10
0
10
20
30
40
50
55
Number Of Gene
up-regulated
down-regulated

B

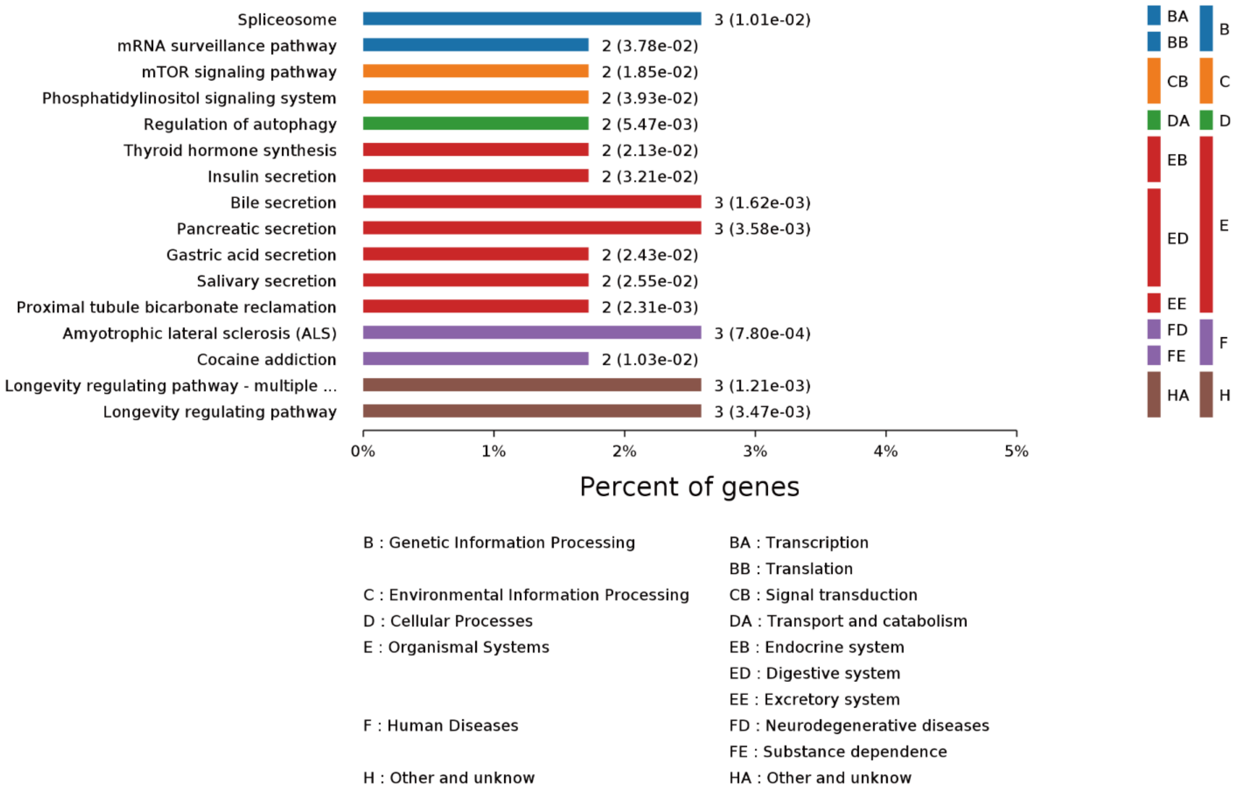
Spliceosome
mRNA surveillance pathway
mTOR signaling pathway
Phosphatidylinositol signaling system
Regulation of autophagy
Thyroid hormone synthesis
Insulin secretion
Bile secretion
Pancreatic secretion
Gastric acid secretion
Salivary secretion
Proximal tubule bicarbonate reclamation
Amyotrophic lateral sclerosis (ALS)
Cocaine addiction
Longevity regulating pathway - multiple ...
Longevity regulating pathway
3 (1.01e-02)
2 (3.78e-02)
2 (1.85e-02)
2 (3.93e-02)
2 (5.47e-03)
2 (2.13e-02)
2 (3.21e-02)
3 (1.62e-03)
3 (3.58e-03)
2 (2.43e-02)
2 (2.55e-02)
2 (2.31e-03)
3 (7.80e-04)
2 (1.03e-02)
3 (1.21e-03)
3 (3.47e-03)
0%
1%
2%
3%
4%
5%
Percent of genes
BA
BB
CB
DA
EB
ED
EE
FD
FE
HA
B
C
D
E
F
H
B : Genetic Information Processing
C : Environmental Information Processing
D : Cellular Processes
E : Organismal Systems
F : Human Diseases
H : Other and unknow
BA : Transcription
BB : Translation
CB : Signal transduction
DA : Transport and catabolism
EB : Endocrine system
ED : Digestive system
EE : Excretory system
FD : Neurodegenerative diseases
FE : Substance dependence
HA : Other and unknow

C

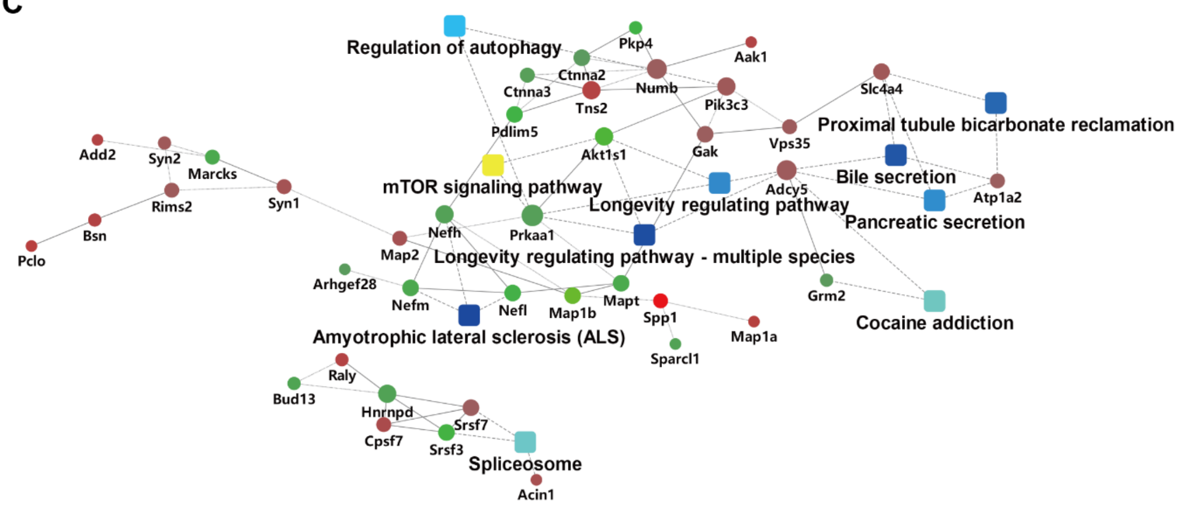
Regulation of autophagy
Pkp4
Aak1
Ctnna2
Numb
Ctnna3
Tns2
Pik3c3
Slc4a4
Pdlim5
Proximal tubule bicarbonate reclamation
Gak
Vps35
Add2
Syn2
Marcks
Akt1s1
Bile secretion
Rims2
Syn1
mTOR signaling pathway
Adcy5
Atp1a2
Bsn
Longevity regulating pathway
Pancreatic secretion
Pclo
Nefh
Prkaa1
Map2
Longevity regulating pathway - multiple species
Arhgef28
Grm2
Mapt
Nefm
Nefl
Map1b
Spp1
Map1a
Cocaine addiction
Amyotrophic lateral sclerosis (ALS)
Sparcl1
Raly
Bud13
Hnrnpd
Srsf7
Cpsf7
Srsf3
Spliceosome
Acin1

Fig.S2 A Differentially expressed genes (DEGs) corresponding to phosphopeptides. **B** KEGG pathway analysis of DEGs. **C** Protein-protein interaction networks of DEGs.

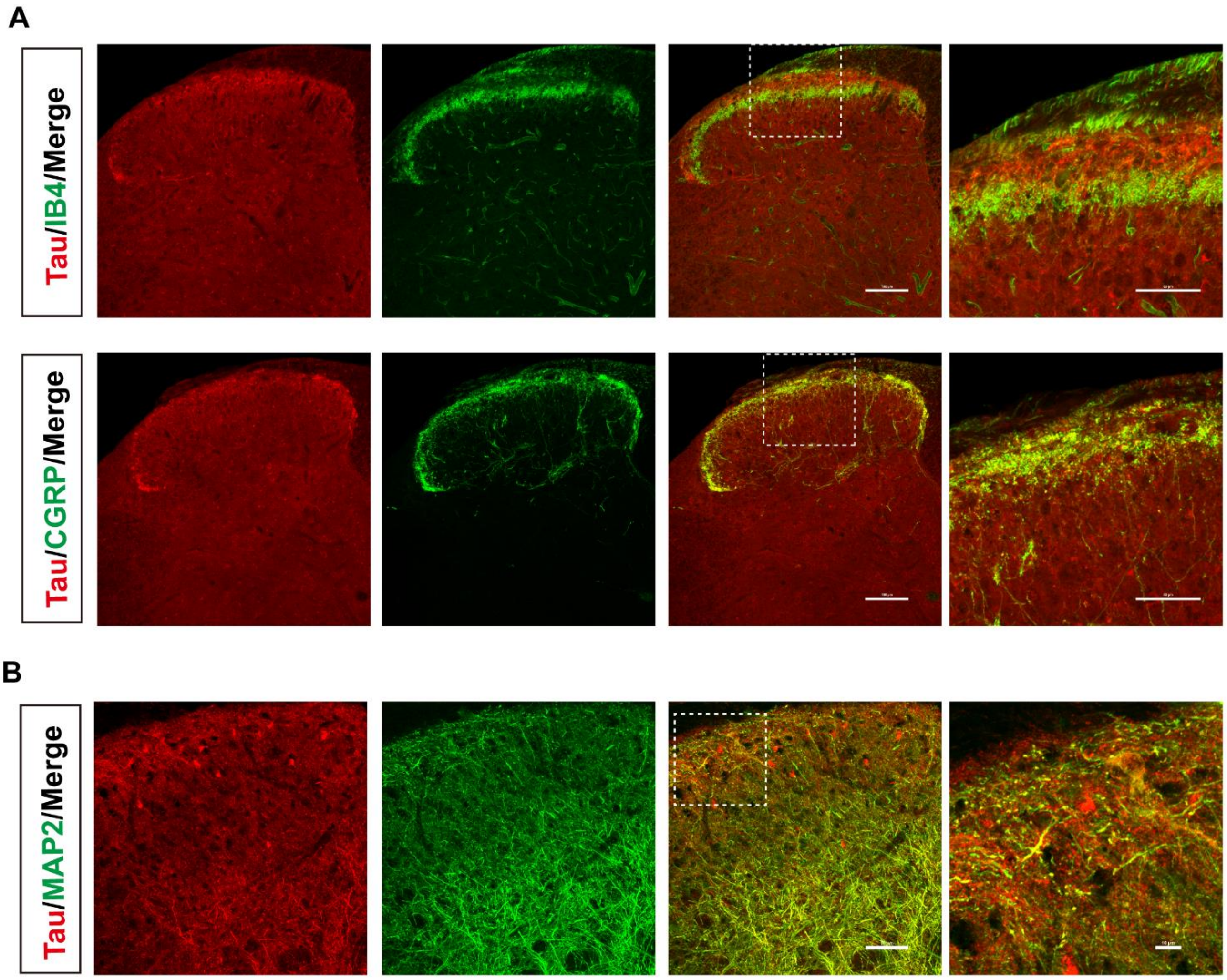

Fig. S3 A Representative images displaying the colocalization of Tau (red) with either IB4 or CGRP (green) in the dorsal cord on day 7 post-CFA injection. Scale bar, 50 μm and 100 μm. **B** Representative images displaying the colocalization of Tau (red) with MAP2 (green) in the dorsal cord on day 7 following CFA injection. Scale bars, 10 μm and 50 μm.

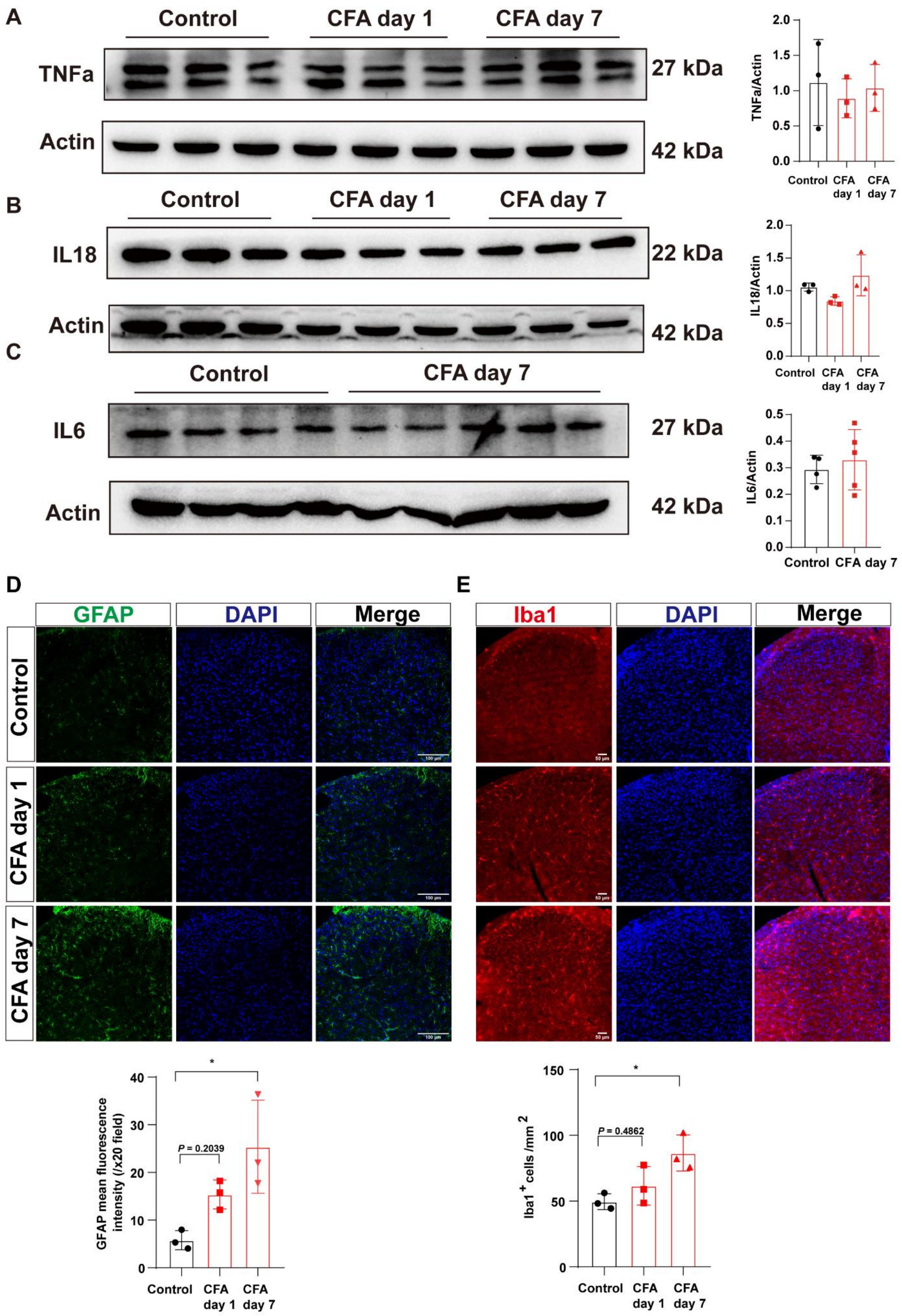
A
Control
CFA day 1
CFA day 7
TNFa
27 kDa
Actin
42 kDa
TNFa/Actin
Control
CFA day 1
CFA day 7
B
IL18
22 kDa
Actin
42 kDa
IL18/Actin
C
IL6
27 kDa
Actin
42 kDa
IL6/Actin
D
GFAP
DAPI
Merge
E
Iba1
DAPI
Merge
P = 0.2039
GFAP mean fluorescence intensity (/x20 field)
P = 0.4862
Iba1+ cells /mm2

Fig. S4 A Western blots and analysis of the protein levels of TNFa in L4–6 in the control group and 1 day and 7 days after CFA injection, normalized to Actin (*n* = 3 per group, one-way ANOVA followed by Tukey's multiple comparisons test). **B** Western blot analysis Protein levels of IL18 of L4–6 in the control group and days 1 and 7 after CFA injection, normalized to Actin (*n* = 3 per group, one-way ANOVA followed by Tukey's multiple comparisons test). **C** Protein levels of IL6 of L4–6 in the control group and day 7 after the CFA injection (*n* = 4 for the control group, *n* = 5 for the CFA day 7 group, unpaired *t* test). **D** Immunofluorescence staining of GFAP (green) in the dorsal cord of L4–6 (*n* = 3 from 3 mice per group, *P <0.05, one-way ANOVA followed by Tukey's multiple comparisons test). **E** Immunofluorescence staining of Iba1 (green) in the dorsal cord of L4–6. Blue, DAPI staining (*n* = 3 from 3 mice per group, *P <0.05, one-way ANOVA followed by Tukey's multiple comparisons test). Scale bars, 50 μm and 100 μm.

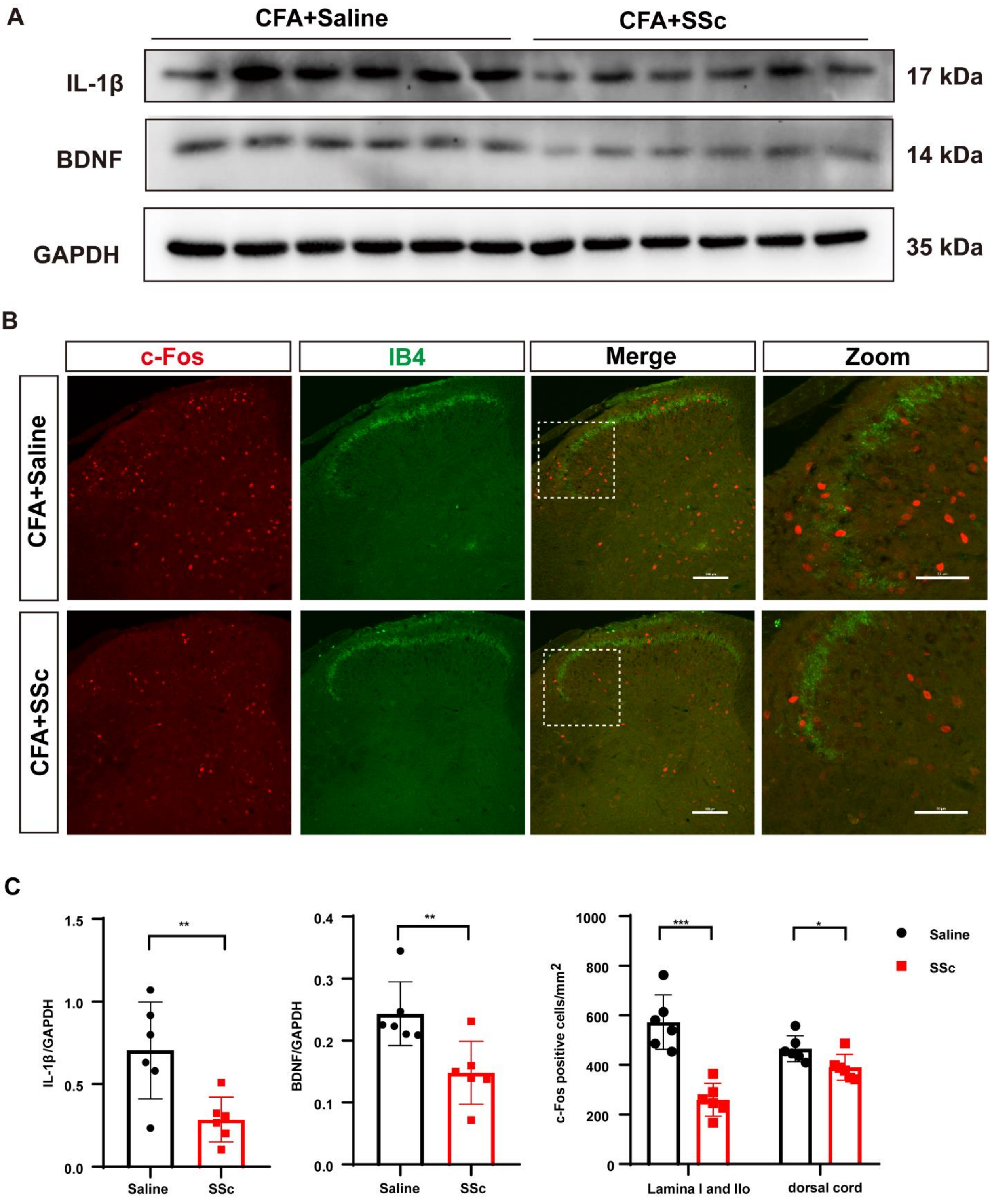

Fig. S5 A Protein levels of IL-1β and BDNF of L4–6 in the CFA + saline group and CFA + SSc group. Western blot analysis, normalized to GAPDH (*n* = 6 per group, unpaired *t* test). **B** Representative immunofluorescence image showing the colocalization of c-Fos and CGRP in the dorsal cord in the

CFA + Saline and CFA+ SSc groups (n = 6 per group, *P <0.05, ***P <0.001, unpaired t-test). **C** Statistical analysis corresponding to the data presented in **A** and **B**. Scale bars, 50 μm and 100 μm.

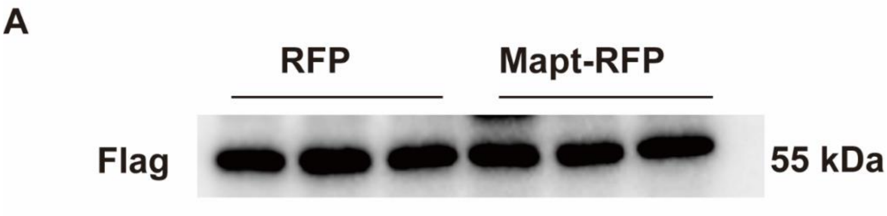

Fig. S6 A Western blots of protein expression of the fusion protein Flag in the AAV-CMV-RFP group and the AAV-CMV-Mapt-RFP group.
